# Testicular Germ Cell Tumor Presenting to the Emergency Department

**DOI:** 10.7759/cureus.12618

**Published:** 2021-01-11

**Authors:** Jose Rubero, Jenny Lee, Matthew Solomon, Jesse C Wu, Latha Ganti

**Affiliations:** 1 Emergency Medicine, University of Central Florida College of Medicine, Orlando, USA; 2 Emergency Medicine, Brown University, Providence, USA; 3 Emergency Medicine, University of Central Florida College of Medicine/HCA Healthcare Graduate Medical Education Consortium Emergency Medicine Residency Program of Greater Orlando, Orlando, USA; 4 Emergency Medicine, Osceola Regional Medical Center, Kissimmee, USA; 5 Emergency Medicine, Envision Physician Services, Plantation, USA; 6 Emergency Medicine, HCA Healthcare Graduate Medical Education Consortium Emergency Medicine Residency Program of Greater Orlando, Orlando, USA

**Keywords:** germ cell tumor, testicular cancer

## Abstract

The authors report on a case of a patient who presented to the emergency department (ED) and was ultimately diagnosed with stage IV testicular non-seminomatous germ cell tumor. The patient was cachectic with a tumor on the neck, abdomen, and scrotum. Germ cell tumors (GCTs) exhibit characteristic symptoms at different points in development. Appropriate treatment can cure most GCTs. While cancer may not be thought of as an ED diagnosis, it can often be the place where patients first present, even when advanced. Recognizing it is important for prompt treatment.

## Introduction

Approximately 95% of malignant testicular neoplasms are of germ cell origin and known as germ cell tumors (GCTs). It is the most common solid malignancy in men aged 15-35 years, even though it only accounts for one percent of all cancers in men [1]. Invasive GCTs are classified as seminomatous and non-seminomatous. Among the non-seminomas, there are five types, including embryonal carcinomas, teratocarcinomas, teratomas, choriocarcinomas, and mixed type [2]. Non-seminomas typically produce several placental and fetal proteins, such as human chorionic gonadotropin (hCG) and alpha-fetoprotein (AFP), which can help in the differential diagnosis [3].

Typically, these tumors will present as an enlarging testicular mass with or without pain. Other symptoms may include pain at sites beyond the scrotum if the cancer is metastasized or gynecomastia from hormonal secretions [4,5]. For all GCTs, initial treatment usually involves orchiectomy. Seminomas are exquisitely sensitive to radiation while non-seminomas are less so [6]. Thus the choice radiation and chemotherapy following orchiectomy depend on the GCT type and stage. Overall the prognosis for GCTs is excellent in the early stages, and seminomas rarely metastasize.

## Case presentation

This is the case of a 30-year-old male that was sent by his primary care physician (PCP) for evaluation of abdominal distention and left-sided neck swelling. The patient had cerebral palsy and was a poor historian. His sister took him to his PCP because he was not eating well, losing weight, and had some shortness of breath. The patient’s PCP noticed an abdominal mass as well as a left-sided neck mass. Neither the patient nor his sister was aware when these symptoms appeared or how long they had been there. The patient had been having normal bowel movements; no abdominal pain, nausea, vomiting, or diarrhea; no chest pain; no fever, chills, cough, or any other complaints. 

On physical examination, he was tachycardic and cachectic with bitemporal wasting. On the left side of the neck, there was a solid golf ball size mass, which was non-movable. The chest evaluation revealed clear breath sounds and tachycardia. Abdominal examination revealed distention with a large palpable grapefruit-sized mass. There was some guarding but no rebound. Genitalia examination showed a hard, non-tender mass of the right scrotum.

Laboratory analysis revealed a white blood cell count of WBC 14.08 K/mm^3^, hemoglobin 10.1 gm/dL, a normal basic metabolic panel, aspartate transaminase (AST) 241 units/L, and alkaline phosphatase 1033 unit/L. We decided to add a beta-hCG because of the testicular mass. It was elevated at 5692 mIU/ml.

**Figure 1:**
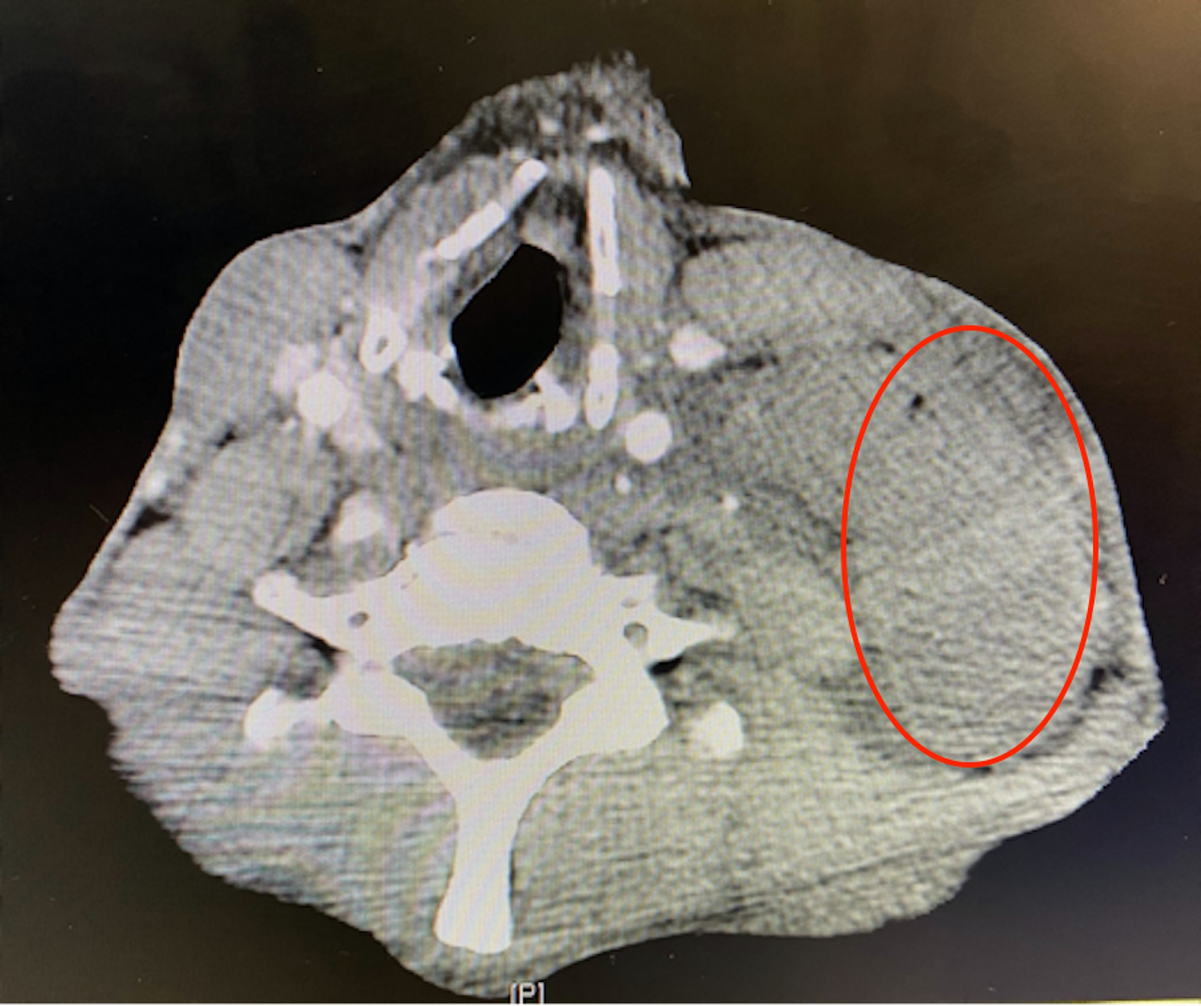
CT demonstrating left-sided neck solid mass (red circle) CT, Computed tomography.

**Figure 2:**
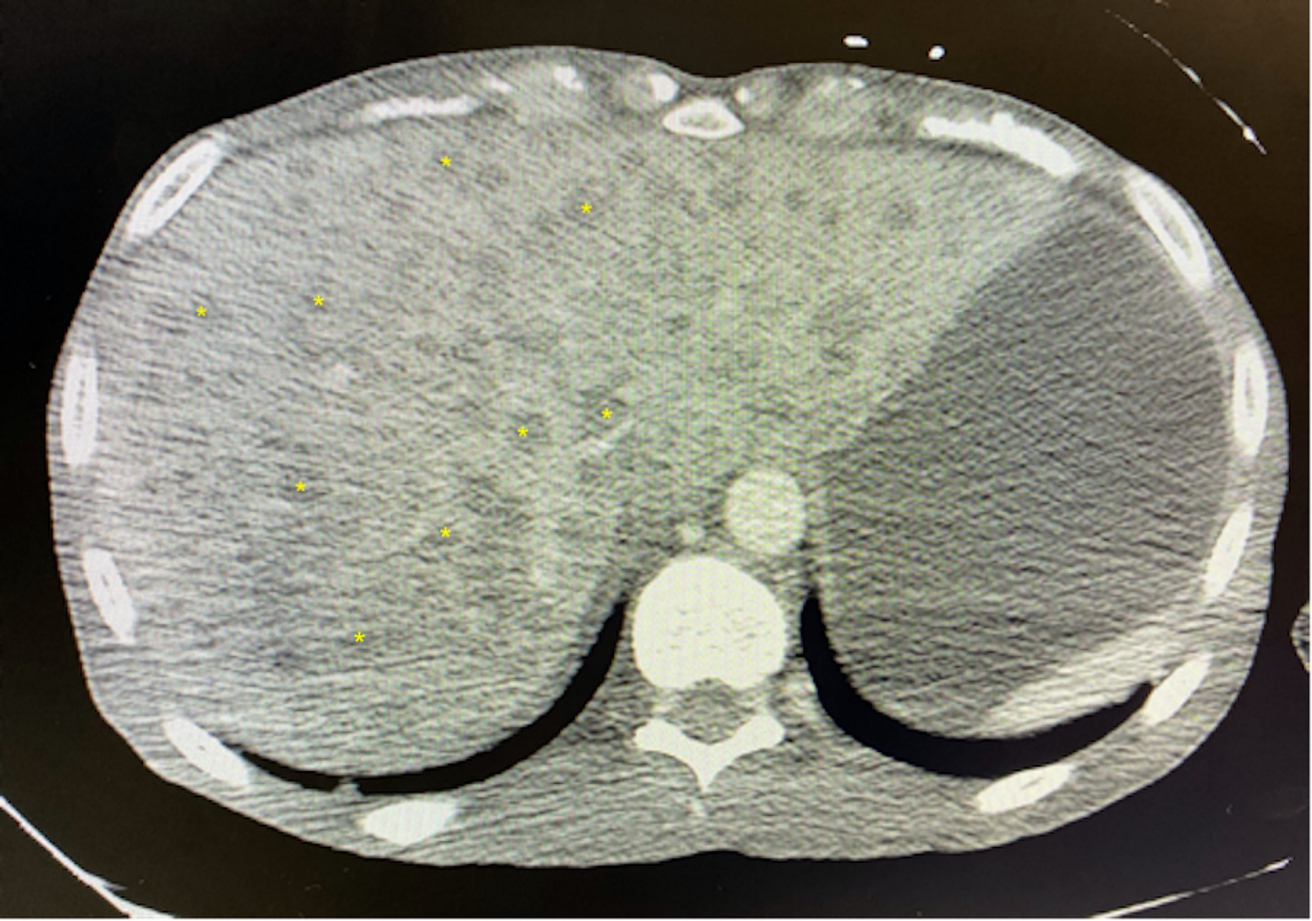
CT scan showing multiple liver nodules and masses (yellow asterisks) CT, Computed tomography.

**Figure 3:**
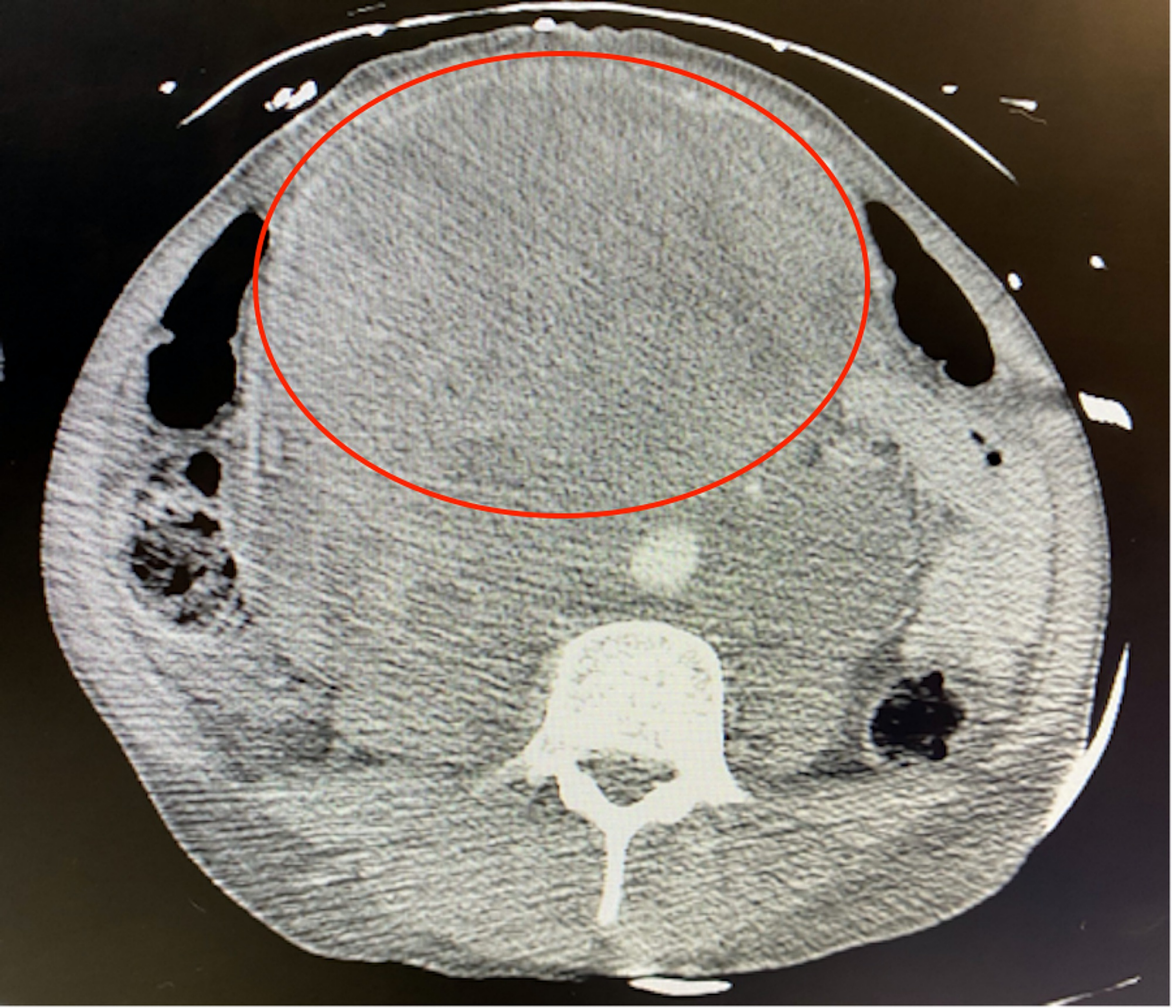
CT scan showing large abdominal mass (red circle) CT, Computed tomography.

**Figure 4:**
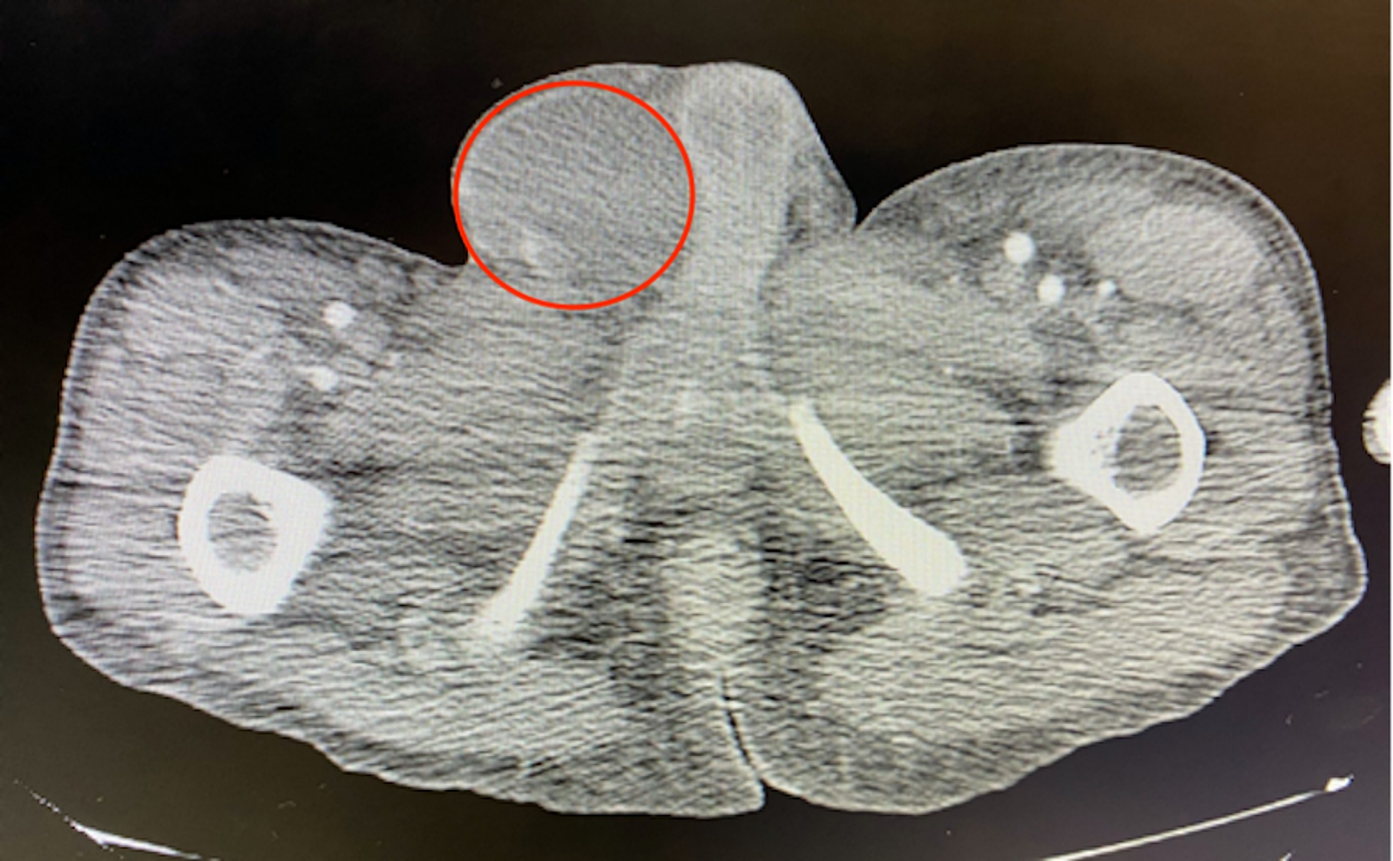
CT scan showing large right testicular mass (red circle) CT, Computed tomography.

Computed tomography (CT) scans of the neck, chest, abdomen, and pelvis showed a large left neck mass (8.6 cm x 4.5 cm) with mediastinal lymphadenopathy versus metastatic lesions; multiple pulmonary nodules and masses; multiple liver and splenic lesions with retroperitoneal lymphadenopathy; large pelvic mass that extends from the level of the liver to the pelvic inlet (17 cm x 13 cm); large retroperitoneal mass that extends from the diaphragmatic hiatus to the aortic bifurcation (19 cm x 10 cm) resulting in mass effect to the abdominal viscera and vasculature; and a pancreatic head mass (5.9 cm x 3.7 cm) (Figures 1-4).

A testicular ultrasound showed no torsion but a solid mass on the right testicle, which was also noted on CT. The patient was admitted to the hospital and underwent orchiectomy. Biopsy of the left neck showed metastatic undifferentiated necrotizing malignant tumor consistent with germ cell tumor with features favoring embryonal carcinoma component. Biopsy of the right testicular mass showed embryonal carcinoma with abundant necrosis and small foci suggestive of yolk sac component. The spermatic cord margin of resection was positive for tumor. The tunica vaginalis was uninvolved. Lymphovascular invasion was present. AFP level was high at 12,200 ng/ml. The patient was diagnosed with stage IV non-seminomatous germ cell tumor (NSGCT) and received four cycles of cisplatin chemotherapy.

## Discussion

**Figure 5:**
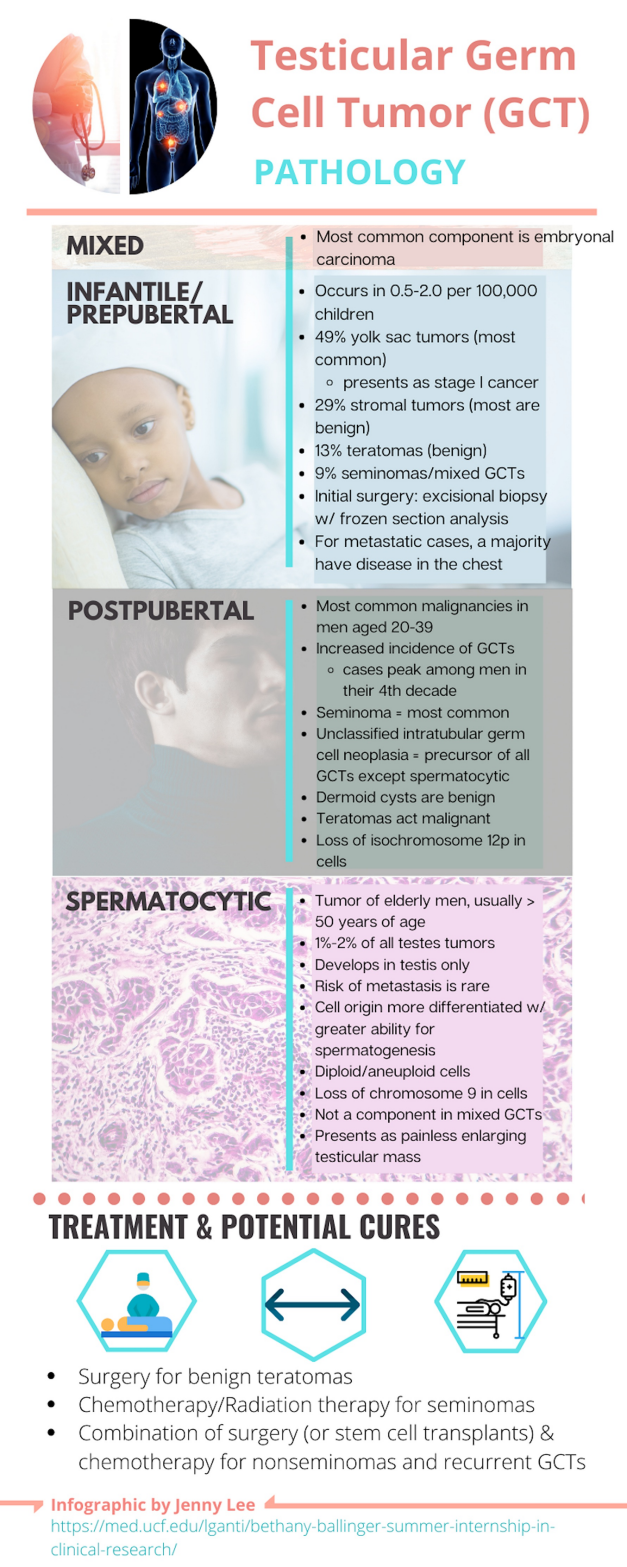
Testicular germ cell tumor pathology

The etiology of GCTs is largely unknown. Cytogenetic studies suggest a different pathogenesis for each group of infantile/prepubertal GCTs, postpubertal GCTs, and spermatocytic tumor (Figure 5).

When patients, especially young adult male, present to the emergency department (ED) with testicular complaints, it is important to keep testicular GCTs on the differential diagnosis. Testicular GCTs are often found incidentally since they are often painless. Patients typically present with a painless solid mass in the scrotum and are well appearing. It is rare for a patient to present with metastatic disease like this case. It may be that his cerebral palsy made history taking challenging, or that his lack of complaining delayed the diagnosis up to this point. There is usually no history of trauma or infection. Other differentials to consider include testicular torsion, Fournier’s gangrene, traumatic testicular rupture, incarcerated inguinal hernia, epididymitis, orchitis, testicular abscess, hydrocele, and varicocele. Testicular ultrasound is the initial diagnostic testing of choice in the ED as it can quickly narrow down a diagnosis. CT scans may be ordered to further characterize the mass and for staging if GCTs are suspected. Patient with suspected GCTs will need urology consultation for orchiectomy and staging. Further management is guided by the staging of GCTs and interdisciplinary team collaboration.

Among GCTs, seminoma is most common. It does not occur before the age of five years. Embryonal carcinoma is the most common component in mixed GCTs. Eighty percent or more of embryonal carcinoma component and vascular invasion are recognized predictors of occult metastasis for clinical stage I mixed GCTs. The most common pediatric GCT is prepubertal yolk sac tumor, which is usually stage I disease at presentation. Most choriocarcinomas present with metastatic symptoms because of the propensity for rapid hematogenous dissemination. Teratomas in children regardless of maturity and dermoid cysts in adults are benign; in contrast, teratomas in adults exhibit malignant behavior. With appropriate therapy, the majority of testicular GCTs are curable [3,7].

Pathology analysis of the tumor following orchiectomy will stratify the GCTs into seminoma and NSGCTs. Seminomas are usually slow-growing, localized tumors and rarely (less than 5%) spread beyond the retroperitoneal nodes [8]. Distant metastasis, like this patient, occurs more frequently with NSGCTs. In addition, seminomas normally do not have elevated beta-hCG or AFP. The patient’s diagnosis of NSGCT is consistent with this. 

For seminomas, radical orchiectomy is usually curative (stage I) while the addition of radiotherapy may be necessary for stage II disease. In the rare event that there is extensive retroperitoneal nodal involvement of distant metastasis, cisplatin-based chemotherapy may be required. Management of NSGCTs depends on the staging that includes a combination of active surveillance, retroperitoneal lymph node dissection (RPLND), and/or chemotherapy. For stage 1 NSGCTs with low risk of relapse post-orchiectomy, generally active surveillance is sufficient, while patients with more advance stage and/or higher risk of relapse may require a combination modality stated above [9]. Unfortunately, our patient had stage IV NSGCT.

## Conclusions

A systematic history and physical examination remain the mainstay of good medicine even in the emergency department. This case illustrates the diagnosis of testicular germ cell tumor that had clearly been present for a while but only picked up after the patient had presented to medical attention a few times. Testicular GCTs once correctly diagnosed can be treated according to the specific type.
